# INSIGHT - INTERVENTION and SCREENING for GENERAL HEALTH in INITIAL PSYCHOSIS TREATMENT (A Clinical Audit)

**DOI:** 10.1192/bjo.2026.11685

**Published:** 2026-06-30

**Authors:** Dipanshu Kakkar, Sarah Kakhi

**Affiliations:** Hampshire and Isle of Wight NHS Foundation Trust, Basingstoke, United Kingdom

## Abstract

**Aims:**

Physical health monitoring is crucial in early psychosis patients because of enhanced risk for physical health issues due to disease and prescribed anti-psychotic treatments. Early monitoring and intervention help identify and manage these risks, improving long term outcomes and quality of life. Physical health is often neglected in mental health care, auditing ensures comprehensive, Integrated care. Addressing physical health can enhance treatment adherence, reduce hospitalizations and improve recovery rates overall.

**Methods:**

We are proposing introduction of Lester UK tool and monitoring and intervening for physical health issues based on Lester UK recommendations only in high-risk patients identified from the case load of mid and north EIP.

High risk patients will be put into Red category

Inclusion criteria:

1. Previous cardiometabolic issues (high BP, cardiac morbidity)

2. High Lipid, HbA1c profile

3. High BMI

4. Smoking

5. Health behaviour and lifestyle changes eg. Alcohol intake Interventions and advice on daily exercise will be offered and will be according to the recommendations set by NCAP(National Clinical audit of Psychosis) adaptation of Lester UK and NAS (National Audit of Schizophrenia)

The standards will be compared with the physical health monitoring standards set by **National Audit of Schizophrenia 2021 and National Clinical Audit for Psychosis 2022.** 1st cycle will be Retrospective to compare local with national standards. Subsequent cycles will be concurrent. Estimated sample size is approx. 30 high risk cases.This will be **reaudited every 6 months** to match the standards. A **target of 80 %** is set to be achieved after the interventions offered.

Retrospective data was collected for first cycle of audit using Blood test results on ICE, Physical health check form on RiO notes and GP data from CHIE/GP Connect. 118 patients were audited and out of these patients 54 have been identified in high-risk zones based on 6 different categories.

**Results:**

Standards were compared with Physical health monitoring and intervention in National Audit of Schizophrenia and National Clinical audit of psychosis.

Monitoring and identifying risk of smoking, alcohol, illicit substance, high BMI, High blood pressure, high lipids and high blood glucose is more than required standards of 75 %.

Acceptance rate of smoking cessation (18%) is significantly below the matching standards of 75 %. This requires proactive measures to educate patients of smoking related health risks and encouraged to quit smoking.

33 % patients accepted NICE recommended interventions for alcohol use, below 75% standards. This requires improved motivational strategies and personalised intervention plans.

Only 6 % patients accepted advise to abstain from illicit substances. This requires motivation and encouragement for patients to refer to Inclusion services and accept advise provided.

100 % patients with high HBs-668C were referred to primary services but only 8 % patients accepted high HAB1C related NICE recommendations. Consistent monitoring and oroactivemanagement of metabolic health is required.

100 % patients from those identified with high Cholesterol/HDL ratio accepted referral to primary services but only 10 % accepted lifestyle modification advice. Ongoing patient education and encouraging for healthy lifestyle is required.

100 % of those identified with high BMI accepted NICE recommendations for healthy life style and referral to primary services. The success of interventions will depend on patient’s adherence and strategies to enhance motivation, and accessibility should be put in place.

100% patients accepted NICE recommended interventions for hypertension and referral to primary services.

**Conclusion:**

INSIGHT Audit reveals gaps in meeting the standards set by NAS and NCAP for interventions although meeting standards for monitoring. While interventions are aligned with national standards, patient engagement and adherence remain major challenges that require action like patient’s education regarding physical health benefits and team’s knowledge and implementation of Lester tool recommended interventions. We have recommended the action plan for interventions before next cycle of audit.

